# *“I was waiting for my period”*: Understanding pregnancy recognition among adolescents seeking abortions in Ethiopia, Malawi, and Zambia^☆,☆☆^

**DOI:** 10.1016/j.contraception.2023.110006

**Published:** 2023-03-15

**Authors:** Joe Strong, Ernestina Coast, Tamara Fetters, Malvern Chiweshe, Abrham Getachew, Risa Griffin, Luke Tembo

**Affiliations:** a London School of Economics and Political Science, Department of Social Policy, London, UK; b London School of Economics and Political Science, Department of International Development, London, UK; c IPAS, Chapel Hill, NC, USA; d Critical Studies in Sexualities and Reproduction, Rhodes University, Makhanda, South Africa; e St. Paul’s Hospital Millennium Medical College, Addis Ababa, Ethiopia; f Risa Griffin Consulting LLC, MN, USA; g Malawi Mission, Addis Ababa, Ethiopia

**Keywords:** Abortion, Adolescents, Pregnancy, Pregnancy recognition, Sexual and reproductive health

## Abstract

**Objectives:**

For a person seeking an abortion, the ability to recognize a pregnancy is a critical first step. Pregnancy recognition is complex and shaped by numerous factors. This paper explores the experiences of pregnancy recognition among adolescents in Ethiopia, Malawi, and Zambia.

**Study design:**

The final sample included 313 adolescents aged 10 to 19 who had sought abortion-related care at urban public facilities in Ethiopia (N = 99), Malawi (N = 104), and Zambia (N = 110). Researchers collected mixed-method data on how adolescents came to recognize that they were pregnant and thematically analyzed qualitative data alongside descriptive statistics from quantitative data.

**Results:**

Most adolescents reported that their main mode of recognizing a pregnancy was medical pregnancy tests or late menstruation. Reasons for not recognizing a pregnancy included irregular menses or recent menarche and attribution of signs and symptoms to other medical conditions. Psychological barriers to pregnancy recognition were important, including the refusal to accept a pregnancy and denial of a pregnancy. Timing of recognition shaped the abortion care available for adolescents and the affordability of care. For some adolescents, their capacity to recognize their pregnancy led to involuntary or voluntary disclosure, which decreased their reproductive autonomy.

**Conclusions:**

Adolescent experiences of pregnancy recognition complement existing evidence, illustrating critical barriers across age and context. Interrogating pregnancy recognition among adolescents exposed the critical implications for the availability, accessibility, affordability, and autonomy of their abortion trajectory.

**Implications:**

Pregnancy recognition is complex and can influence adolescents’ ability to exercise their reproductive rights and access abortion care of their choosing. Programmes to improve awareness of the signs of a pregnancy, increasing the provision of affordable and accessible pregnancy testing and further research on pregnancy recognition are necessary to support adolescents’ reproductive autonomy.

## 1 Introduction

For all pregnancy-related care, pregnancy recognition is the critical first step. Pregnancy recognition is complex, nuanced, and multifaceted [1,2]. Seeking abortion care is time-sensitive, and the timing of pregnancy recognition can have a significant impact on the accessibility, affordability, and availability of abortion care [3]. Despite the importance of pregnancy recognition, it is rarely researched in detail to understand the factors that can facilitate or delay recognition [2]. Adolescents seeking abortions frequently recognize their pregnancies later than adults, yet research on pregnancy recognition has tended to focus on dating gestational ages among primarily adult abortion care-seekers [4–8].

Factors that lead to pregnancy recognition include late or missed menstruation, contraceptive nonuse or failure, bodily changes beyond menstrual changes, such as breast tenderness or nausea [9–12]. However, an individual’s experiences shape these factors, such as prior irregular menstruation, psycho-social responses, including belief in not being at risk of pregnancy or denial of pregnancy, and the ‘intendedness’ of a pregnancy [1,8,11–17]. For adolescents, these individual factors can be exacerbated by age-related inequalities and stigma that affect the accessibility of sexual and reproductive health services [18,19]. Adolescents can be less likely to seek pregnancy tests, to acknowledge menstrual changes, and to be psychologically prepared to recognize a pregnancy compared to adults [13,15,20–23]. This makes it especially important to understand the experiences of pregnancy recognition among adolescents.

Ethiopia, Malawi, and Zambia represent three distinct health system, legal and policy approaches to abortion (see Table 1) [24]. In each country, adolescents represent a significant proportion of pregnancies that end in abortion. In Ethiopia, it is estimated that 20% of pregnancies among adolescents aged 15 to 19 end in an abortion [25]. In Malawi, an estimated third of adolescents aged 15 to 19 and a fifth aged 12 to 14 reported that a friend had tried to end a pregnancy [26]. In Zambia, evidence from government hospitals found that a quarter of people seeking abortions were adolescents aged 15 to 19 [27]. In all three countries, a significant proportion of women and girls seek abortions after 12 weeks [28–31].

This paper seeks to understand the intersecting factors that comprise adolescent pregnancy recognition and the implications for abortion experiences. It provides insights into the complexities of pregnancy recognition among adolescents aged 10 to 19 years who sought abortion-related care in Ethiopia, Malawi, and Zambia.

## 2 Methods

This study is drawn from a larger comparative, cross-country research project in Ethiopia, Malawi, and Zambia, conducted between 2018 and 2019. The research team designed the study to gather data on adolescent abortion trajectories, from the circumstances surrounding their pregnancy, to their experiences of abortion-related care. Table 1 outlines the availability of abortion and gestational limits (where applicable) in these countries.

The study included two public facilities in each country; one referral hospital and one facility designated as “youth friendly.” Reflecting country-level legal differences in the provision of safe abortion services for adolescents, the study sites in Ethiopia and Zambia each included a public referral hospital and a youth friendly district-level public health facility, and site selection was informed by available data on the volume of abortion-related care-seeking by facility. In Malawi, a public maternity care referral hospital and a nongovernmental designated youth friendly sexual and reproductive health clinic were selected by the research team as recruitment sites, reflecting the highly restrictive legal context of abortion and that in the public sector most abortion-related care is for postabortion care.

A study-trained senior nurse identified adolescents for recruitment based on the criteria of either having sought either an abortion or postabortion care (treatment following an abortion). The senior nurse invited adolescents to participate in the study once ready for discharge; nurses were not involved in research consent procedures. Trained female research assistants completed informed consent with all participants, dependent on whether the participant was a minor (below 18 years) or an adult. Research assistants sought consent for minors either from an accompanying parent or guardian (with the minor’s assent) or from the minor themselves in cases where they had sought care without a parent/guardian and were treated as an “emancipated minor.”

Two research assistants interviewed each adolescent simultaneously, collecting qualitative and quantitative data for all adolescents. The first interviewer conducted a semistructured interview, which was recorded to generate qualitative data. The second interviewer completed a datasheet during the interview, which gathered quantitative data, and asked follow-up questions not covered by the first interviewer. This approach reduced the overall interview time for each adolescent and meant that qualitative data and quantitative data could be matched through respondent IDs for mixed-method analysis. The research instruments are available (https://abortioninafrica.wordpress.com/). Research assistants conducted interviews in local languages. The research assistants completed verbatim translation and transcription of recorded interviews, with quality checks.

Team members (J.S., E.C., T.F., M.C., R.G.) cleaned the quantitative data, and reviewed unclear responses against the interview transcripts to ensure consistency and accurate data capture. Using Dedoose, we coded qualitative data and analyzed using a combination of deductive and inductive themes to explore issues related to adolescent abortion and contraceptive care-seeking and decision-making. We collaboratively developed the codebook, included blind coding of 10 cases by all analyzers to check for intercoder variability and understandings. Three members of the team (E.C., M.C., T.F.) double coded 49% of the interviews to check for internal consistency; the remaining interviews were single coded.

The authors determined ‘abortion safety’ (safe versus less or least safe) using biomedical classifications developed by Ganatra et al. [32] and classified for each participant based on their interview evidence and medical records (where available). ‘Safe’ abortions were those for which adolescents received medically recommended care within a clinical setting. Self-use of medication abortion was categorized as ‘less safe’ and was based on adolescent’s descriptions of their experiences and the drugs they used. Nonmedical abortion pharmaceuticals and self-management with toxins, objects inserted in the uterus, or herbal abortifacients were all categorized as ‘least safe.’

Ethical review was obtained in Ethiopia (Ethiopian Public Health Institute: 154-2018), Malawi (National Health Sciences Research Committee: 2003), Zambia (ERES-2017-Nov-005), and the UK (London School of Economics: 000606).

### 2.1 Reflexivity

Adolescents’ responses might reflect socio-desirability tied to the legality of abortion in their context, as well as the power differentials between adolescents and interviewers. The two-interviewer approach, in which the first interviewer used a conversational and informal style of interview while the second interviewer took notes and asked probing or follow-up questions to complete the datasheet, was used to create a more conversational interview [27]. We designed this approach to create an environment in which adolescents were more comfortable to disclose and share information about their abortion-related care-seeking, compared to a standard survey interview.

We represent researchers from the Global North and South, and we conducted the analysis with varying knowledge of the specific study context. It is possible during the translation and analysis process that contextual nuances were underdeveloped. We sought to account for these in the double-coding protocol. Double coding of 49% of transcripts led to the iteration and development of a code-book that reflected the breadth of research team experiences and pluralities of epistemological backgrounds. This process allowed for more nuances that might otherwise have been missed in a codebook developed by a single researcher.

## 3 Results

### 3.1 Sample description

A total of 313 interviews were conducted (Ethiopia N = 99; Malawi N = 104; Zambia N = 110). Most adolescents in Ethiopia obtained a safe abortion, whereas most adolescents in Zambia and almost all adolescents in Malawi obtained a less or least safe abortion (Table 2).

### 3.2 Factors leading to pregnancy recognition

Research assistants asked adolescents how they first thought they might be pregnant, with follow-up prompts to capture any additional factors, signs, or symptoms of pregnancy recognition. Research assistants recorded responses on the datasheet through a table of primary indicators and open text for additional factors.

Nearly half of adolescents reported that their primary indicator of pregnancy recognition was a medical pregnancy test (urine test, ultrasound, or other), followed by late/missed menstruation (Table 3). Four adolescents in Zambia reported not knowing they were pregnant, though their interviews showed they had sought an abortion elsewhere. Disaggregating by age, older adolescents (18 years and above) more frequently reported using a pregnancy test compared to younger adolescents, who were more likely to report late menstruation.

Data on the costs of pregnancy tests and other modes of pregnancy recognition indicate the potentially prohibitive costs for adolescents. A small number of adolescents provided information on associated costs in Ethiopia (n = 27 [27.3%]) and Malawi (n = 28 [26.9%]); a researcher training error meant that this information was not collected in Zambia. Only one (1%) adolescent in Ethiopia and four (3.8%) in Malawi reported that they were able to access a medical pregnancy test for free. Costs varied considerably; between approximately zero and 16 USD in Ethiopia and zero and seven USD in Malawi. Adolescents paid on average under one USD in both Ethiopia and Malawi.

For many adolescents, pregnancy recognition was a result of a culmination of different factors involving missed or late periods, alongside seeking information from a third party (a friend, family member, or pharmacist), before obtaining a test:

When my period was late and my belly got bigger I suspected that I might be pregnant. A girl that I work for … suspected that I might be pregnant

19-year-old, Ethiopia, abortion at the recruitment facility, nulliparous

Many adolescents indicated multiple factors culminating in their recognition of their pregnancy, with 228 (72.8%) citing between two and four factors. Of these, 216 (69%) of adolescents cited that late menstruation was a factor leading to pregnancy recognition and 201 (64.2%) reported a pregnancy test, while 136 (43.5%) adolescents did not report a medical pregnancy test at any point.

### 3.3 Factors that delayed pregnancy recognition

Adolescents gave several reasons why they did not rely on a single factor, sign, or symptom in recognizing their pregnancy. Many adolescents had multiple missed periods before recognizing a pregnancy. Particularly in Ethiopia, some adolescents bought multiple medical pregnancy tests, increasing the cost of pregnancy recognition:

At home, I tested my urine and there were two lines, but the other one was not clear to identify. So, I bought the testing kit three times.

19-year-old, Ethiopia, abortion at the recruitment facility, nulliparous

Prior irregular menstruation, as well as recent menarche, meant adolescents did not connect changes in menstruation to a pregnancy:

Int: As you told me before, your pregnancy was more than three months, why were you late to terminate the pregnancy?

R: Because I did not know about my pregnancy. I was waiting for my period, but it did not happen as usual.

18-year-old, Ethiopia, abortion at the recruitment facility, nulliparous

Not recognizing that signs or symptoms could be associated with a pregnancy was a major contributor to delayed recognition. For some adolescents, symptoms such as vomiting (n = 58 [18.5%]), breast tenderness (n = 30 [9.6%]), and nausea (n = 29 [9.3%]) were commonly reported but not understood as signs of a pregnancy. For others, the association of symptoms with other conditions (e.g., malaria, typhoid) limited the extent to which adolescents reported that they suspected they could be pregnant:

Since I was sick by malaria before, I thought it was malaria this time too.

18-year-old, Ethiopia, abortion at the recruitment facility, nulliparous

Delays to pregnancy recognition were also connected with psychological barriers. For some adolescents, fear of pregnancy meant that despite being aware that they could be pregnant, they delayed confirmation:

My boyfriend told me to do a test, but I didn’t do a test since I was scared. I went to a health centre for two days and back to my home without doing anything. I was afraid to ask the health providers about the pregnancy test.

19-year-old Ethiopia, abortion at the recruitment facility, given birth once before

For other adolescents, they did not want to believe that they could be pregnant:

The first time I tested, I believed that it was a lie.

17-year-old, Zambia, post-abortion care at the recruitment facility after taking pills to induce abortion, nulliparous

Adolescents described how the belief that they could not get pregnant delayed their pregnancy recognition. In some cases, this was linked to a sexual partner assuring them that they could not get pregnant because he used a condom or would withdraw, or it was their first time having sex. For some adolescents, the nature of the sexual encounter was a factor in their assumptions, for example, not knowing or believing that their partner had ejaculated, or not thinking that pregnancy could result from the first sexual encounter:

R: I didn’t know that I was pregnant… I didn’t think that I would be pregnant with one sex. My period was delayed for two months.

19-year-old, Ethiopia, post-abortion care at the recruitment facility after taking misoprostol at home, nulliparous

### 3.4 Impacts of pregnancy recognition on abortion trajectories

The factors that impact delayed pregnancy recognition intersect to intensify the barriers to care and the choices available. Where care should have been available, the process of pregnancy recognition allowed for significant delays and obstacles, including being refused care by an abortion provider due to nonpolicy-based gestational age restrictions:

I went to [referral facility] while my period got late for three consecutive months. The health providers in [referral facility] scanned with ultrasound and told me that the termination service not given for the pregnancy for more than 2 months.

18-year-old, Ethiopia, abortion at recruitment facility, nulliparous

Where there was limited choice of abortion methods due to higher gestational ages, there were also significant cost implications for adolescents. This meant that pregnancy recognition had a critical impact on the affordability of an abortion and, therefore, the facilities accessible to adolescents:

First, they [private hospital staff] tested me, it was positive, they conducted a scan then they said the baby is four months… That’s when the amount of money which the doctor said it was too much, that’s when my mother took me here [to the study facility].

15-year-old, Zambia, abortion at the recruitment facility, nulliparous

Adolescents also discussed how their pregnancy recognition had implications for the autonomy of their pregnancy and abortion decision-making. The involvement of third parties was both sought and sometimes received when not desired. Some adolescents used people around them to determine whether they were pregnant or help explain why they were experiencing certain symptoms. These people subsequently were involved in their abortion decision-making:

I missed my monthly period for a month. So, when I told my friend that I have missed my monthly period, she told me that I was pregnant and we should do that we should terminate the pregnancy

17-year-old, Malawi, post-abortion care at the recruitment facility after taking misoprostol at home, nulliparous

For other adolescents, delayed recognition of a pregnancy led to uninvited interventions from others—particularly female friends and relatives. This undermined the ability of adolescents to make autonomous choices about their pregnancies and directly impacted decisions to obtain an abortion:

R: She [her mother] is the one that noticed that I was pregnant and when she asked me, I did not say anything. So, she said we should go for a pregnancy test and when we got there, and they found that I was pregnant. My mother said we should abort the pregnancy.

15-year-old, Malawi, post-abortion care at the recruitment facility after taking misoprostol at home, nulliparous

This adolescent’s mother was able to directly influence the decision to obtain an abortion by recognizing her daughter’s pregnancy first. While disclosure might facilitate the abortion an adolescent wanted, the nature of disclosure, the experience of disclosure, and the choice and voice of the adolescent in deciding their pregnancy outcome could be diminished.

Third-party involvement, particularly that of health care workers, was also a factor in the process of obtaining medical pregnancy tests. This partly reflected country-level differences, as adolescents in Ethiopia were more likely to confirm their pregnancy at a pharmacy or other medical/clinical setting than the other two countries. Some adolescents reported that pregnancy testing through medical clinics could necessitate multiple visits and delay their access to abortion care. For some, interactions with test providers could be critical in obtaining information for abortion access:

I told them that my period was late for two months. Then they did a urine test and told me that I was pregnant. I was really upset at that time when I heard from the nurse who did a urine test. Then she [the nurse] told me to go to the doctor for the service [abortion] I needed to get.

18-year-old. Ethiopia, abortion at the recruitment facility, nulliparous

Adolescents also described how interactions during pregnancy recognition—particularly through buying pregnancy tests—were linked to obtaining abortion information and nonfacility-based abortion care from health care workers:

I suspected I was pregnant. So, my cousin and I went to a drug store, and they tested me for pregnancy. I was told that I was pregnant. I asked them what I would do, and they told us to come the next day. They said they sold medicine used to terminate pregnancy for K450 [approximately 26 USD].

15-year-old, Zambia, post-abortion care at the recruitment facility after inserting pills into her vagina, nulliparous

The highly restrictive abortion laws in Malawi, coupled with lower rates of medical pregnancy testing, meant that these interactions between adolescents and health care workers were far less frequent.

## 4 Discussion

Recognition of a pregnancy is the critical first step in any abortion trajectory [3]. Pregnancy recognition is complex, dynamic, and can have a significant impact on an adolescent’s abortion care. Country and age differences suggest structural factors that shape pregnancy recognition, including the accessibility of pregnancy tests and abortion in each context [24]. Pregnancy recognition intersects with existing barriers for adolescents to accessing sexual and reproductive health services, including costs of tests, lack of privacy, and information barriers [33,34]. Adolescents in this study faced similar barriers identified in studies with adult populations across different contexts [1,5,6,35,36]. Adolescents’ experiences were shaped by historically irregular menstruation/recent menarche, psychological factors, and their sexual experience and partner [1,12,14–16,37].

This study highlights how pregnancy recognition is implicated in abortion choice, access, timeliness, and disclosure among pregnant adolescents. Adolescents made explicit how their pregnancy recognition could include a constellation of actors—pharmacists, partners, relatives, and work colleagues—involved in their abortion-related care [38]. For some, this was the consensual involvement of informal support networks to confirm their pregnancies and discuss their decision-making, including health care workers that provide pregnancy tests. This research shows that experiences of pregnancy recognition can undermine the tenets of reproductive autonomy and choice among adolescents who are forced to disclose.

Our sample is comprised of adolescents with the agency and/or resources to navigate pregnancy recognition and seek an abortion or postabortion care; our approach excludes adolescents unable to obtain abortions or postabortion care, or those that did not need postabortion care. Additional insights into potential sequencing patterns in recognition and specific timings were not captured, nor could precise estimations of gestational age be made. We were not able to disaggregate between different types of pregnancy tests, such as urine tests and ultrasounds. As an exploratory study, our mixed-method approach allows for descriptive and generative insights into adolescent pregnancy recognition. This study cannot be statistically generalized to adolescent experiences but aims to generate insights on pregnancy recognition [39].

Facilitating pregnancy recognition will allow for the mitigation of negative experiences and barriers that lead to delays in abortion care-seeking. This includes the provision of accessible and affordable pregnancy tests, accounting for the specific need to respect adolescents’ rights. Efforts to increase knowledge on potential signs—and causes—of a pregnancy are important, including acknowledgment of different menstrual experiences and how they impact pregnancy recognition among adolescents. The inclusion of pregnancy recognition in comprehensive sexuality education can help increase adolescent awareness of the signs of a pregnancy. Further research on pregnancy recognition is critical to understanding how recognition intersects with abortion trajectories and the barriers to safe and accessible abortions to adolescents who want them.

## Figures and Tables

**Table 1:** Abortion policies in Ethiopia, Malawi, and Zambia at the time of data collection in 2018/19

	Ethiopia	Malawi	Zambia
Conditions under which abortions are permitted	Life, mental and physical health, of pregnant woman; rape and incest [without requiring evidence]; mental or physical disability including due to minority status of pregnant woman; fetal impairment. Includes provision to terminate pregnancies legally on the grounds of being below the age of 18 without requiring proof of age.	Life of pregnant woman	Life, mental and physical health of pregnant woman; physical and mental health of existing children; fetal impairment; adolescents under 16
Restrictions based on gestational age	28 weeks from last normal menstrual date for any indication Assessments of gestational age include estimating last normal menstrual date; physical finding (abdominal and pelvic examination); and ultrasound (optional)	No gestational age limit specified for exemption	‘Viability’ used as limit, considered to be 28 weeks
Availability of safe abortion services	Available in the public, private, and NGO sectors, depending on gestational age and method	Very limited availability	Some availability in public sector facilities; limited availability in the private/ NGO sector

Source: Global Abortion Policies Database [24,40].

**Table 2:** Characteristics of sample of adolescents in Ethiopia, Malawi, and Zambia (2018/19)

	Ethiopia (n = 99) n (%)	Malawi (n = 104) n (%)	Zambia (n = 110) n (%)
**Medical safety of abortion**			
Safe	97 (98)	4 (4)	37 (34)
Less or least safe	2 (2)	100 (96)	73 (66)
**Age**			
10–14	0 (0)	4 (3.8)	12 (10.9)
15–17	28 (28.3)	61 (58.7)	52 (48.2)
18–19	71 (71.7)	39 (37.5)	45 (40.9)
**Currently in school**			
Yes	39 (39.4)	70 (67.3)	79 (71.8)
**Highest current education**			
Primary or below	62 (62.6)	60 (57.7)	13 (11.8)
Secondary or above	37 (35.4)	42 (40.4)	96 (87.3)
No answer	2 (2)	2 (1.9)	1 (0.9)
**Occupation**			
No school or work	25 (25.3)	22 (21.2)	22 (20)
School and work	12 (12.1)	6 (5.8)	4 (3.6)
School, no work	27 (27.3)	61 (58.7)	72 (65.5)
Work, no school	35 (35.4)	8 (7.7)	6 (5.5)
**Paid work**			
Yes	47 (47.5)	14 (13.5)	10 (9.1)

**Table 3:** Primary indicator of pregnancy for adolescents, by country

Primary indicator of pregnancy	Country Ethiopia (n = 99) n (%)	Malawi (n = 104) n (%)	Zambia (n = 110) n (%)
Late menstruation	34 (34.3)	60 (57.7)	35 (31.8)
Someone else noticed	9 (9.1)	4 (3.8)	10 (9.1)
Medical pregnancy test	51 (51.5)	33 (31.7)	60 (54.5)
Body changes	5 (5.1)	7 (6.7)	1 (0.9)
Other	0	0	4 (3.6)
